# The association between serum cadmium and diabetes in the general population: A cross-sectional study from NHANES (1999–2020)

**DOI:** 10.3389/fnut.2022.966500

**Published:** 2022-12-07

**Authors:** Rongpeng Gong, Xiaolu Pu, Zhenqian Cheng, Jie Ding, Zhenghao Chen, Yongjun Wang

**Affiliations:** ^1^Qinghai University, Xining, Qinghai, China; ^2^Department of Clinical Nutrition, The First Affiliated Hospital of Shandong First Medical University and Shandong Provincial Qianfoshan Hospital, Jinan, Shandong, China; ^3^University of Electronic Science and Technology of China, Chengdu, Sichuan, China; ^4^National Institute for Nutrition and Health, Chinese Center for Disease Control and Prevention, Beijing, China

**Keywords:** diabetes, serum cadmium, U.S. adults, cross-sectional study, NHANES

## Abstract

**Background:**

Associations between serum cadmium and diabetes had been reported in previous studies, however there was still considerable controversy regarding associations. Studies in general population that investigated the effects of serum cadmium on diabetes were currently lacking. We designed this cross-sectional study among U.S. adults under high and low cadmium exposure to assess associations between serum cadmium and diabetes.

**Methods:**

This cross-sectional study analyzed 52,593 adults who aged more than 20 years and participated in the National Health and Nutrition Examination Survey (NHANES), 1999–2020. The missing values and extreme values in the covariables were filled by multiple interpolation. Univariate logistics regression, multivariate logistics regression and smooth fitting curves were used to analyze the association between serum cadmium and diabetes. Simultaneously, sensitivity analysis was carried out by converting the serum cadmium from continuous variable to categorical variable. The stratification logistics regression model was used to analyze whether there were special groups in each subgroup to test the stability of the results.

**Results:**

In this cross-sectional study, serum cadmium levels were negatively correlated with the occurrence of diabetes in the low serum cadmium exposure group (OR = 0.811, 95% CI 0.698, 0.943; *P* = 0.007). There was no association between serum cadmium level and the occurrence of diabetes in the high serum cadmium exposure group (OR = 1.01, 95% CI 0.982, 1.037; *P* = 0.511). These results were consistent across all the subgroups (*P* for interaction >0.05).

**Conclusion:**

Serum cadmium was negatively associated diabetes among the representative samples of the whole population in the United States under the normal level of serum cadmium exposure. However, there was no association between serum cadmium level and the occurrence of diabetes in the high serum cadmium exposure group. This study promoted an update of new preventative strategy targeting environment for the prevention and control of diabetes in the future.

## Introduction

Cadmium is a widespread industrial and environmental pollutant (1). Routes of cadmium exposure include breathing, eating, or drinking the substance, or by skin contact with food and tobacco being the primary sources of exposure in the United States (2–6). Cadmium and its compounds are highly toxic to human body and can be accumulated, causing damage to different systems and tissues and organs of the body (7, 8). Cadmium can enter human body and spread through the blood, mainly accumulated in the liver and kidney, followed by the thyroid, spleen, pancreas and other organs (9, 10). Long-term exposure to high-level cadmium can lead to damage to respiratory organs, impaired renal function, impaired immune system function, metabolic disorders, endocrine disorders and other symptoms (11–15). However, researches on the harm of cadmium exposure is mainly based on high exposure level, but the low exposure level is limited.

With the development of economy, diabetes has gradually become one of the major non-communicable diseases endangering the health of people all over the world (16–18). According to International Diabetes Federation (IDF) statistics, the global diabetes prevalence was estimated at 10.5% in adults and by 2045 the estimated number of diabetes will have increased by 46% (19). Diabetes and its complications lead to increased All-cause mortality and disability-adjusted life-years (DALYs) in individuals with diabetes (20). Diabetes has become one of the most severe and critical public health problems (18, 21). Therefore, it is urgent to prevent and control the occurrence and development of diabetes.

The pathogenic factors of diabetes are multifactorial, in addition to the traditional risk factors of diabetes, such as genetic susceptibility, abdominal obesity, unhealthy diet and lifestyle and so on (22–24), various studies have shown that environmental factor is a significant risk factor for the occurrence of diabetes, severe environmental pollution (18, 25–28). Given cadmium as one of the sources of heavy metal pollution, people paid more and more attention to the effect of cadmium exposure on the development of diabetes in recent years. Previous epidemiological studies have suggested that cadmium exposure is closely related to the occurrence and development of diabetes. Studies suggest a positive association between elevated cadmium concentrations and diabetes risk (28, 29). In contrast, some study results indicated that diabetes risk decreased with cadmium exposure (30). Additionally, two studies (31, 32) did not find any associations between cadmium exposure and diabetes risk. Taken together, these results suggest that conclusions from studies are somewhat inconsistent and the mechanism remain unclear. In addition, amounting studies have been performed studies limited to pregnant women, infants and occupational cadmium exposed workers (33–35), whereas the general population is likely affected by cadmium exposure in the form of low concentration and long-term exposure. Therefore, it is necessary to explore the association between serum cadmium and diabetes risk in the general population.

Therefore, in our study we aimed to investigate if cadmium exposure was associated with diabetes in general adults and determine if the association presented in a dose-response manner under different levels of cadmium exposure.

## Method

### The data source

The National Health and Nutritional Health Survey (NHANES) is an extensive cross-sectional survey conducted nationwide. It is an extensive multiagency program designed to monitor adults' and children's the health and nutritional status across the United States (36). The survey is unique in its combination of interviews and detailed physical examinations. The multi-stage stratified probability design was adopted in the population sampling of the survey, to make the sample representative of the entire American population.

The data set consisted of demoinformatics, body measurement, laboratory, and questionnaire data. In this study, the data set of 11 cycles (1999–2020) in the NHANES database of the National Center for Health of the United States were selected (37). Meanwhile, the data of 11 cycles were standardized consolidation according to the National Center for Health Statistics (NCHS) recommendations by using interview weights.

More information from NHANES can inquire on the website (https://www.cdc.gov/nchs/nhanes/index.htm), and other places have similar reports.

### Study design and participants

This study is a large cross-sectional study of adults in the United States. The independent variable was the participants' serum cadmium levels, and the target dependent variable was whether the participants were diagnosed with diabetes. Participants were divided into two groups according to their serum cadmium levels. The participants in the low cadmium exposure group were lower than 75% of the serum cadmium levels of all participants, and the participants in the high cadmium exposure group were higher than 75% of the serum cadmium levels of all participants (38). Then, the association between serum cadmium and diabetes in the two groups was discussed, respectively.

**Figure 1:**
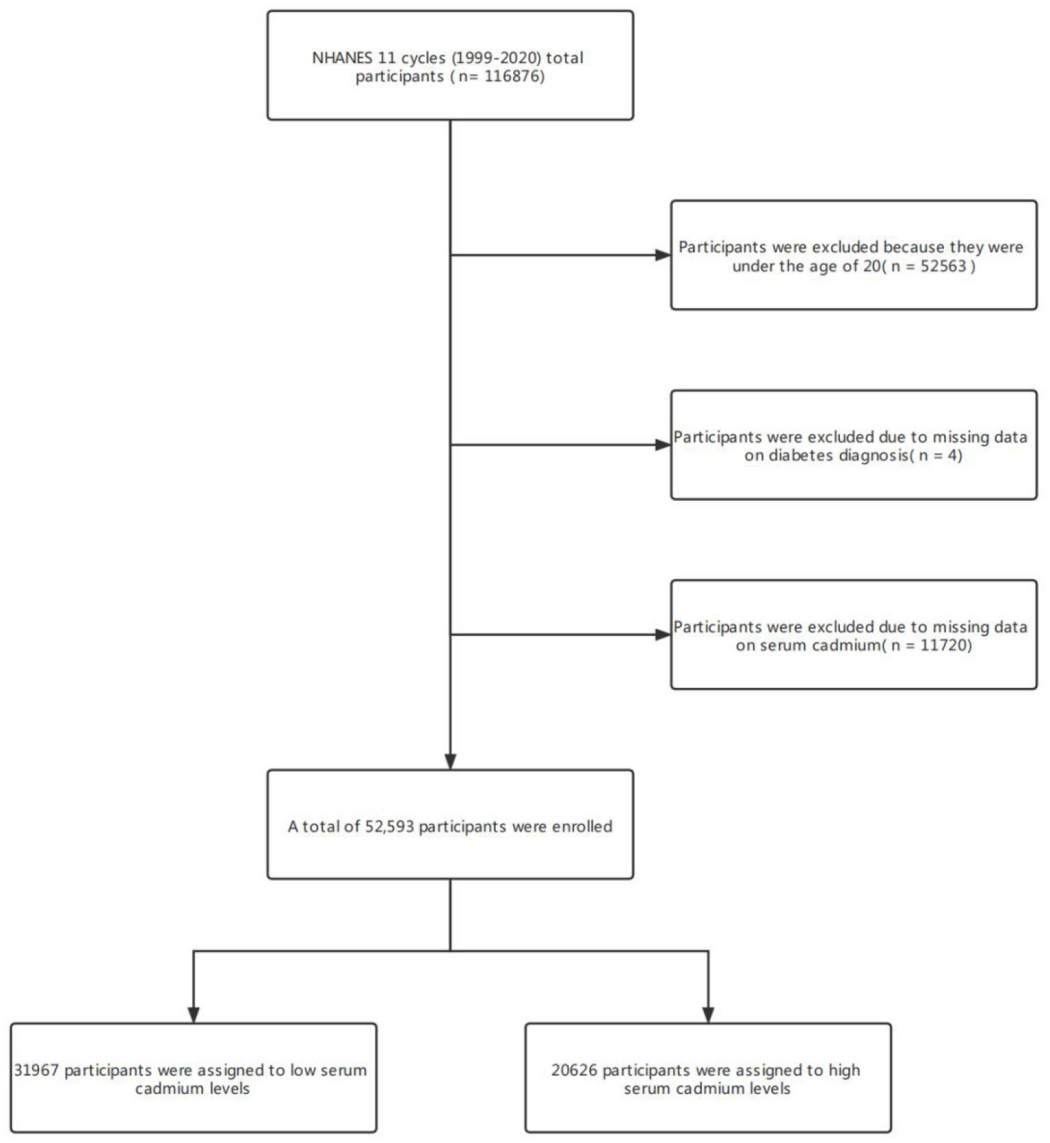
Flowchart of participant selection.

116,876 participants were surveyed in the NHANES project between 1999 and 2020. This study established strict inclusion and exclusion criteria to exclude people who do not meet this study. The criteria were as follows: (1) participants younger than 20 years of age (*n* = 52563); (2) Participants who did not undergo serum cadmium testing (*n* = 11720); (3) Participants who lacked diagnostic data for diabetes (*n* = 4). Finally, 52,593 participants were enrolled. 31,967 participants were assigned to the low cadmium exposure group and 20,626 to the high cadmium exposure group (Figure 1).

The study was approved by the National Research Ethics Review Board for Health Statistics and received written informed consent from all participants. Specific information about the ethical review can be found on the NHANES website.

### Data collection

All data in this study were collected and recorded by uniformly trained investigators after qualified visits. The data used included demographics (age, sex, race, education level, etc.), anthropometry [waist circumference (WC), body mass index (BMI), etc.], health-related behaviors (smoking, alcohol consumption, etc.), and biochemical indicators [millimeters.high-density lipoprotein(HDL); triglyceride(TG); alanine aminotransferase(ALT); γ-glutamyl transpeptadase(GGT); lactate dehydrogenase(LDH); albumin(ALB), etc.]. Basic information was collected immediately by investigators, and biochemical samples were stored and managed scientifically and sent to the Laboratory Science Division of the National Center for Environmental Health of the Centers for Disease Control and Prevention (Atlanta, GA, USA), the Laboratory of the University of Minnesota and the University of Missouri-Columbia for testing and analysis.

#### Measurement of serum cadmium

Professional personnel collected blood samples during the physical examination and froze at −20°C after collection. Blood samples were sent to the Laboratory Science Division of the National Center for Environmental Health, Centers for Disease Control and Prevention (Atlanta, GA, USA) for analysis and treatment. The collected and stored materials for metal analysis were pre-screened for background contamination prior to analysis. Serum cadmium was analyzed using a Perkin Elmer SIMAA 6000 instrument. In addition, the quantitative detection of cadmium is based on the measurement of light absorbed at 228.8 and 283.3 nm, respectively by ground state atoms of cadmium from an electroless discharge lamp (EDL) or hollow cathode lamp (HCL) source.

#### Diagnosis of diabetes

The diagnosis of diabetes is made in combination with guidelines issued by the International Diabetes Association (IDM) and the clinical criteria currently in use. Diabetes can be determined if it meets any of the following criteria: (1) participants with fasting blood glucose ≥7.0 mmol/L in laboratory tests; (2) Participants with blood glucose >11.1 mmol/L in OGTT experiment; (3) Participants who were taking diabetes medications; (4) Participants who were diagnosed with diabetes by their doctors during the survey; (5) Participants who self-reported being diagnosed with diabetes.

#### Other variables

In NHANES, some sociodemographic information is collected by structured data. Here, participants comprised three categories according to their level of education. The lower level of education: education level below grade 11, and secondary education: high school graduate or equivalent and people with higher education: college or higher education. Participants were also divided into three groups according whether they smoked or not. Participants were considered current smokers if they had smoked 100 or more cigarettes in the past and reported smoking several days or daily at the time of the interview. Participants who had smoked fewer than 100 cigarettes in the past but did not currently smoke were considered former smokers, and participants who had fewer than 100 cigarettes in their past were considered nonsmokers. Alcohol consumption is based on the 2015–2020 Dietary Guidelines for Americans issued by the U.S. Departments of Health and Human Services and Agriculture. Male drinkers were defined as had more than two drinks per day, and female drinkers had more than one drink per day. The racial breakdown was based on the time of the survey: Mexican Americans, non-Hispanic whites, non-Hispanic blacks, other Hispanics, and other races. Among the participants, the diagnosis of hypertension was based on the ISH2020 International Hypertension Practice Guidelines published by International Society of Hypertension (ISH) and commonly used clinical criteria (39): (1) participants' average blood pressure (tested twice or more) or participants' blood pressure (tested only once) had SBP >140 mmHg or DBP >90 mmHg; (2) Participants were taking hypertension medications. (3) Participants were diagnosed with hypertension during follow-up; (4) Participants who self-reported being diagnosed with hypertension. BMI is calculated based on height and weight. Its calculation formula is BMI = weight (kg)/height (m^2^); Height was measured using electronic Sports Measurements with an accuracy of millimeters. Weight was measured by researchers using a digital Scale and converted pounds to kilograms when the measurement was complete. WC was measured using electronic Sports Measurements with an accuracy of millimeters. TG, ALT, GGT, LDH and other biochemical measurements were analyzed by the University of Minnesota laboratory and the University of Missouri-Columbia. All experimental biochemical data were analyzed under quality control. NHANES Quality Control and Quality Assurance Protocols (QA/QC) meet the requirements of the Clinical Laboratory Improvement Act 1988. Detailed QA/QC instructions are discussed in the NHANES LPM. For detailed instructions on quality assurance and quality control procedures, please refer to the NHANES website.

### Statistical methods

The data selected for this study was analyzed by R open-source statistical software, version 4.1.2. Detailed sample descriptions represent continuous variables, and the average confidence interval is 95%, the normal distribution is described by median and standard deviation, skewed distribution is based on median and Q1-Q3. Counts and weighted percentages represent categorical variables. Continuous variables were compared between groups using Mann-Whitney U test or Student *T*-test based on distribution normality. *P* < 0.05(bilateral) was considered statistically significant. Univariate logistics and multivariate logistics regression were used to analyze the association between serum cadmium and diabetes. The selection of covariates in multi-factor logistics regression is based on the variables reported in previous relevant literature, variables that affect the outcome in international expert consensus, variables that change the target dependent variable by more than 10% in single-factor analysis and our relevant clinical experience. The included covariables are as follows: age, sex, race, education, BMI, WC, smoking, alcohol consumption, hypertension, HDL, TG, ALT, GGT, LDH, and ALB. In this study, the missing values and extreme values in the covariables are filled by multiple interpolations. In addition, sensitivity analysis was performed to observe significant differences between the newly generated dataset and the original dataset. However, these studies revealed no significant difference (*P* >0.05). Therefore, according to Rubin's criterion, Our multivariate analysis results are based on the data set after multiple interpolations. Four logistics regression models and smooth fitting curves are constructed in this study. The trend test was carried out by converting the serum cadmium from a continuous variable to a categorical variable. Smooth curve fitting analyses based on logistic regression models of continuous serum cadmium to assess the shape of associations between serum cadmium and diabetes (40). Simultaneously, the subgroup logistics regression model is used to analyze whether there are special groups in each subgroup to test the stability of the results.

## Results

### Basic information description of participants

A total of 52,593 participants were included in this study [Male: 25,348 (48.2%), Female: 27,245 (51.8%)], there were 31,967 in the low serum cadmium exposure group [Male: 16,114 (50.4%); Female: 15,853 (49.6%)], 20,626 in the high serum cadmium exposure group [Male: 9,324 (44.8%); Female: 11,392 (55.2%)]. There was significant difference in gender distribution between the two groups (*P* < 0.001). The mean age of participants was 50.0 ± 18.1 years, and the mean age of the high serum cadmium exposure group was slightly higher than that of the low serum cadmium exposure group (53.6 ± 17.8 vs. 47.7 ± 18.0 years). There were also significant differences between the high and low serum cadmium exposure groups in ethnic education, hypertension, smoking and alcohol consumption (*P* < 0.001). Compared with the low serum cadmium exposure group, BMI (28.1 ± 6.5 vs. 29.7 ± 7.0 kg/m^2^), WC (97.3 ±15.6 vs. 100.1 ± 16.3 cm) and ALT [19.0 (15.0, 26.0) vs. 21.0 (16.0, 28.0) mmol/L] were lower in the high exposure group, GGT [21.0 (15.0, 34.0) vs. 20.0 (14.0, 30.0) mmol/L], LDH [136.0 (119.0, 157.0) vs. 134.0 (117.0, 155.0) mmol/L] and serum cadmium (6.3 (4.8, 9.8) vs. 2.1 (1.4, 2.8) mmol/L) levels were higher. In addition, there were significant differences in the distribution of HDL, TG and ALB levels between the two groups (*P* < 0.001) (Table 1).

**Table 1:** Characteristics of participants according to serum cadmium exposure levels.

**Variables**	**Total** **(*n =* 52593)**	**Low cadmium exposure group (*n =* 31967)**	**High cadmium exposure group (*n =* 20626)**	***P*** **-value**
Age, Mean ± SD	50.0 ± 18.1	47.7 ± 18.0	53.6 ± 17.8	<0.001
Gender, *n* (%)				<0.001
Male	25,348 (48.2)	16,114 (50.4)	9,234 (44.8)	
Female	27,245 (51.8)	15,853 (49.6)	11,392 (55.2)	
Race, *n* (%)				<0.001
Mexican American	8,937 (17.0)	6,152 (19.2)	2,785 (13.5)	
Other Hispanic	11,152 (21.2)	6,467 (20.2)	4,685 (22.7)	
Non-Hispanic white	22,965 (43.7)	13,656 (42.7)	9,309 (45.1)	
Non-Hispanic black	4,387 (8.3)	3,115 (9.7)	1,272 (6.2)	
Other races	5,152 (9.8)	2,577 (8.1)	2,575 (12.5)	
Education, *n* (%)				<0.001
Poorly educated	13,812 (26.3)	7,243 (22.7)	6,569 (31.9)	
Moderately educated	12,286 (23.4)	6,901 (21.6)	5,385 (26.2)	
highly educated	26,416 (50.3)	17,782 (55.7)	8,634 (41.9)	
BMI, Mean ± SD	29.1 ± 6.9	29.7 ± 7.0	28.1 ± 6.5	<0.001
WC, Mean ± SD	99.0 ± 16.1	100.1 ± 16.3	97.3 ± 15.6	<0.001
DM, *n* (%)				0.131
No	43,178 (82.1)	26,179 (81.9)	16,999 (82.4)	
Yes	9,415 (17.9)	5,788 (18.1)	3,627 (17.6)	
Hypertension, *n* (%)				<0.001
Yes	19,162 (36.4)	10,760 (33.7)	8,402 (40.7)	
No	33,420 (63.6)	21,200 (66.3)	12,220 (59.3)	
Smoke, *n* (%)				<0.001
Never smoking	28,728 (54.7)	22,441 (70.2)	6,287 (30.5)	
Former smokers	13,058 (24.8)	7,995 (25)	5,063 (24.6)	
Current smoker	10,766 (20.5)	1,513 (4.7)	9,253 (44.9)	
Alcohol consumption, *n* (%)				<0.001
Yes	9,845 (25.8)	5,734 (24.9)	4,111 (27.1)	
No	28,302 (74.2)	17,259 (75.1)	11,043 (72.9)	
HDL, Median (IQR)	1.3 (1.1, 1.6)	1.3 (1.1, 1.6)	1.3 (1.1, 1.6)	<0.001
TG, Median (IQR)	1.3 (0.9, 2.0)	1.3 (0.9, 2.0)	1.3 (0.9, 2.0)	0.018
ALT, Median (IQR)	20.0 (15.0, 28.0)	21.0 (16.0, 28.0)	19.0 (15.0, 26.0)	<0.001
GGT, Median (IQR)	20.0 (14.0, 32.0)	20.0 (14.0, 30.0)	21.0 (15.0, 34.0)	<0.001
LDH, Median (IQR)	135.0 (118.0, 156.0)	134.0 (117.0, 155.0)	136.0 (119.0, 157.0)	<0.001
ALB, Median (IQR)	42.0 (40.0, 44.0)	42.0 (40.0, 45.0)	42.0 (40.0, 44.0)	<0.001
Cadmium, Median (IQR)	3.2 (1.8, 5.3)	2.1 (1.4, 2.8)	6.3 (4.8, 9.8)	<0.001

HDL, high-density lipoprotein; TG, triglyceride; ALT, alanine aminotransferase; GGT, γ-glutamyl transpeptadase; LDH, lactate dehydrogenase; ALB, albumin; BMI, body mass index; WC, Waist Circumference.

### Univariate logistics analysis of diabetes-related variable

**Figure 2:**
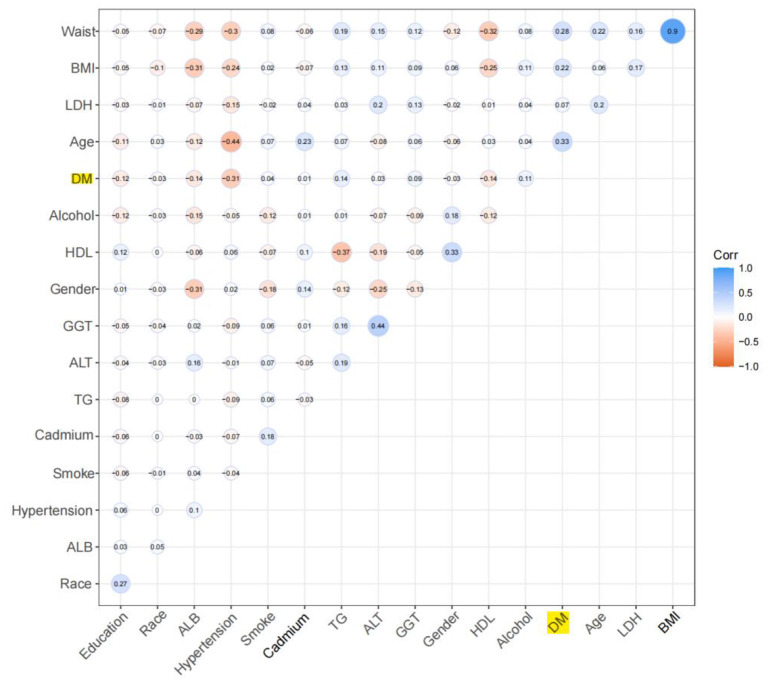
Pearson correlation coefficient plot. With the decrease of Pearson's *r*, the blue in the figure deepens, which means the negative correlation is stronger; with the increase of Pearson's *r*, the red in the figure deepens, which means that the positive correlation is stronger.

Univariate logistics analysis was used for observation of the associations between age, sex, race, education level, BMI, WC, smoking, alcohol consumption, biochemical indicators and the incidence of diabetes before and after multiple interpolation in the US population. In the low serum cadmium exposure group, the associations between variables and diabetes were represented in Table 2 and Figure 2, and the results before and after the interpolation indicated little difference, so we took the results after the interpolation as the final result. We found that age was positively associated with the occurrence of diabetes, and the effect value OR and 95% confidence interval were 1.06 (1.05, 1.07), respectively. Compared with male, female was less likely to develop diabetes, with OR and 95% CI of 0.84 (0.66, 1.06), respectively. Compared with Mexican Americans, other Hispanics [OR: 1.42 (0.86, 2.36)] and other races [OR: 1.30 (0.70, 2.38)] had a higher incidence of diabetes, while non-Hispanic whites [OR: 0.90 (0.60, 1.37)] had a lower incidence of diabetes. Among different levels of education, with low level of education as the reference, the incidence of diabetes was lower in those with high level of education [OR: 0.67 (0.47, 0.95)] and high level of education [OR: 0.45 (0.34, 0.62)]. Compared with non-smokers, former smokers were more likely to develop diabetes, with OR and 95% CI of 1.57 (1.22, 2.02), respectively. Nonalcoholic participants were more likely to develop diabetes than those who consumed alcohol [OR: 1.94 (1.42, 2.65)]. Meanwhile, we found that BMI, WC and some biochemical indicators, such as TG, ALT, GGT and LDH, were positively associated with the occurrence of diabetes, HDL and ALB were negatively associated with the occurrence of diabetes, and serum cadmium was not associated with the occurrence of diabetes (*P* > 0.05).

**Table 2:** Univariate logistic regression analysis for diabetes in the low and high cadmium exposure groups, respectively.

	**Univariate logistic regression analysis for diabetes in the low cadmium exposure group**				
**Variable**	**Before multiple interpolation**		**After multiple interpolation**	
	**OR (95% CI)**	***P*** **-value**	**OR (95% CI)**	***P*** **-value**
(Intercept)	0.16 (0.14–0.18)	<0.001	0.15 (0.13–0.17)	<0.001
Age	1.06 (1.05–1.06)	<0.001	1.06 (1.05–1.07)	<0.001
Gender				
Male	1		1	
Female	0.83 (0.67–1.03)	0.088	0.84 (0.66–1.06)	0.135
Race				
Mexican American	1		1	
Other Hispanic	1.36 (0.86–2.15)	0.184	1.42 (0.86–2.36)	0.173
Non-Hispanic white	0.87 (0.60–1.28)	0.488	0.90 (0.60–1.37)	0.629
Non-Hispanic black	1.01 (0.59–1.73)	0.964	1.02 (0.56–1.86)	0.939
Other races	1.18 (0.68–2.02)	0.558	1.30 (0.70–2.38)	0.405
Education				
Poorly educated	1		1	
Moderately educated	0.69 (0.50–0.96)	0.026	0.67 (0.47–0.95)	0.025
Highly educated	0.48 (0.36–0.63)	<0.001	0.45 (0.34–0.62)	<0.001
HDL	0.27 (0.19–0.37)	<0.001	0.28 (0.19–0.40)	<0.001
TG	1.33 (1.24–1.42)	<0.001	1.32 (1.22–1.42)	<0.001
ALT	1.01 (1.00–1.01)	0.011	1.01 (1.00–1.01)	0.019
GGT	1.01 (1.00–1.01)	<0.001	1.01 (1.00–1.01)	<0.001
LDH	1.01 (1.00–1.01)	<0.001	1.01 (1.00–1.01)	0.002
ALB	0.88 (0.86–0.91)	<0.001	0.88 (0.85–0.91)	<0.001
BMI	1.09 (1.08–1.11)	<0.001	1.10 (1.08–1.11)	<0.001
WC	1.05 (1.04–1.06)	<0.001	1.05 (1.05–1.06)	<0.001
Cadmium	1.01 (0.89–1.14)	0.917	1.02 (0.89–1.17)	0.754
Hypertension				
	1		1	
	0.19 (0.15–0.23)	<0.001	0.18 (0.14–0.24)	<0.001
Smoke				
Never smoking	1		1	
Former smokers	1.57 (1.25–1.97)	<0.001	1.57 (1.22–2.02)	<0.001
Current smoker	0.75 (0.42–1.34)	0.337	0.80 (0.43–1.48)	0.476
Alcohol1 consumption				
Yes	1		1	
No	1.98 (1.51–2.61)	<0.001	1.94 (1.42–2.65)	<0.001

HDL, high-density lipoprotein; TG, triglyceride; ALT, alanine aminotransferase; GGT, γ-glutamyl transpeptadase; LDH, lactate dehydrogenase; ALB, albumin; BMI, body mass index; WC, Waist Circumference.

In the group with high serum cadmium exposure, the association between variables and diabetes was shown in Table 3, and the results before and after the interpolation presented little difference, therefore we took the results after the interpolation as the final result. We found that age was positively correlated with the occurrence of diabetes, and the effect value OR and 95% confidence interval were 1.045 (1.035, 1.054), respectively. There was no difference in the incidence of diabetes between male and female (*P* = 0.735). There was no difference in the incidence of diabetes among ethnic groups. Among different levels of education, with low level of education as the reference, the incidence of diabetes was lower in those with medium level of education [OR: 0.637 (0.439, 0.924)] and high level of education [OR: 0.565 (0.403, 0.793)]. Compared with non-smokers, former smokers were more likely to develop diabetes, with an OR and 95% CI of 1.49 (1.023, 2.172), respectively. Non-alcoholic participants were more likely to develop diabetes than those who consumed alcohol [OR: 2.078 (1.442, 2.994)]. Meanwhile, we found that BMI, WC and some biochemical indicators, including TG, GGT and LDH, were positively correlated with the occurrence of diabetes; HDL and ALB were negatively correlated with the occurrence of diabetes; ALT level and serum cadmium were not correlated with the occurrence of diabetes (*P* > 0.05).

**Table 3:** Univariate logistic regression analysis for diabetes in the high cadmium exposure.

	**Univariate logistic regression analysis for diabetes in the high cadmium exposure group**				
**Variable**	**Before multiple interpolation**		**After multiple interpolation**	
	**OR(95% CI)**	***P*** **-value**	**OR(95% CI)**	***P*** **-value**
(Intercept)	0.012 (0.007–0.022)	<0.001	0.013 (0.007–0.022)	<0.001
Age	1.045 (1.035–1.055)	<0.001	1.045 (1.035–1.054)	<0.001
Gender				
Male	1		1	
Female	0.939 (0.691–1.276)	0.686	0.952 (0.715–1.267)	0.735
Race				
Mexican American	1		1	
Other Hispanic	0.967 (0.475–1.966)	0.925	0.975 (0.497–1.911)	0.941
Non-Hispanic white	0.631 (0.341–1.168)	0.143	0.664 (0.368–1.198)	0.174
Non-Hispanic black	0.832 (0.342–2.024)	0.685	0.902 (0.393–2.071)	0.808
Other races	1.062 (0.504–2.24)	0.874	1.083 (0.539–2.176)	0.822
Education				
Poorly educated	1		1	
Moderately educated	0.631 (0.424–0.938)	0.023	0.637 (0.439–0.924)	0.018
Highly educated	0.553 (0.385–0.794)	0.001	0.565 (0.403–0.793)	0.001
HDL	0.446 (0.291–0.684)	<0.001	0.424 (0.290–0.622)	<0.001
TG	1.247 (1.138–1.366)	<0.001	1.251 (1.147–1.365)	<0.001
ALT	1.001 (0.998–1.005)	0.377	1.002 (0.998–1.005)	0.364
GGT	1.003 (1.001–1.006)	0.012	1.003 (1.001–1.005)	0.009
LDH	1.005 (1.001–1.01)	0.017	1.005 (1.001–1.009)	0.008
ALB	0.888 (0.850–0.928)	<0.001	0.886 (0.851–0.921)	<0.001
BMI	1.084 (1.061–1.108)	<0.001	1.083 (1.062–1.105)	<0.001
WC	1.047 (1.037–1.058)	<0.001	1.046 (1.037–1.055)	<0.001
Cadmium	0.990 (0.966–1.016)	0.449	0.992 (0.969–1.015)	0.499
Hypertension				
Yes	1		1	
No	0.272 (0.198–0.373)	<0.001	0.266 (0.199–0.357)	<0.001
Smoke				
Never smoking	1		1	
Former smokers	1.527 (1.022–2.283)	0.039	1.490 (1.023–2.172)	0.038
Current smoker	0.755 (0.515–1.105)	0.148	0.761 (0.535–1.082)	0.128
Alcohol1 consumption				
Yes	1		1	
No	2.074 (1.366–3.151)	<0.001	2.078 (1.442–2.994)	<0.001

HDL, high-density lipoprotein; TG, triglyceride; ALT, alanine aminotransferase; GGT, γ-glutamyl transpeptadase; LDH, lactate dehydrogenase; ALB, albumin; BMI, body mass index; WC, Waist Circumference.

### Multivariable logistics regression analysis of the association between serum cadmium level and diabetes

In this study, we established four logistic regression models to analyze the relationship between serum cadmium level and diabetes in American adults. OR of the models can be interpreted as the change of serum cadmium level, the probability of developing diabetes also changes accordingly. Table 3 shows the association between serum cadmium and diabetes before and after data interpolation in the low serum cadmium exposure group. For example, in model 2, the effect value OR and 95% CI were 0.745 (0.649, 0.856), respectively, indicating that each unit increase of serum cadmium level reduced the probability of diabetes by 35.5%. The effect value OR and 95% CI of model 1 were 1.007 (0.892, 1.136). The effect value OR and 95% CI of model 3 were 0.798 (0.688, 0.925), respectively. The effect value OR and 95% CI of model 4 were 0.811 (0.698, 0.943), respectively. Here, the results of model 3 and Model 4 are similar, indicating that the adjustment strategy of Model 4 has been quite sufficient. In conclusion, serum cadmium levels were negatively correlated with the diabetes in the low serum cadmium exposure group. Table 4 shows the association between serum cadmium and diabetes before and after data interpolation in the high serum cadmium exposure group. We observed that in all models of the high serum cadmium exposure group, there was no association between serum cadmium level and the occurrence of diabetes, and no statistical significance (all *P* > 0.05) (Table 5). Supplementary Tables S1, S2 respectively revealed the association between serum cadmium level and diabetes in five different interpolation data sets in the low and high serum cadmium exposure groups. In addition, to ensure the stability of the results, trend test was carried out in this study. The serum cadmium level was converted from continuous variable to categorical variable, and quartered according to the quartile of serum cadmium, with Q1 as reference. In the low cadmium exposure group, models 2–4 showed a negative correlation between serum cadmium levels and the occurrence of diabetes, and the relationship was monotonically decreasing (All *P* for trend < 0.05). This indicated that there was a stable and negative correlation between serum cadmium level and the occurrence of diabetes (Supplementary Table S3). In the high cadmium exposure group, all models revealed no association with the development of diabetes when serum cadmium was used as a categorical variable.

**Table 4:** ORs (95%CIs) of the association between serum cadmium levels and diabetes in the low cadmium exposure group.

**ORs (95%CIs) in the low cadmium exposure group**				
**Variable**	**Serum cadmium (Before the interpolation)**		**Serum cadmium (after the interpolation)**	
	**OR (95% CI)**	***P*** **value**	**OR (95% CI)**	***P*** **-value**
Model 1	1.022 (0.892–1.170)	0.750	1.007 (0.892–1.136)	0.910
Model 2	0.754 (0.647–0.88)	<0.001	0.745 (0.649–0.856)	<0.001
Model 3	0.794 (0.663–0.950)	0.011	0.798 (0.688–0.925)	0.003
Model 4	0.801 (0.659–0.974)	0.026	0.811 (0.698–0.943)	0.007

Model 1: non-adjusted.

Model 2: age, gender, race, education.

Model 3: Model 2+ BMI, waist, smolking, alchol consumption, hypertension.

Model 4: Model 3+ HDL, TG, ALT, GGT, LDH, ALB.

**Table 5:** ORs (95%CIs) of the association between serum cadmium levels and diabetes in the high cadmium exposure group.

**ORs (95%CIs) in the high cadmium exposure group**				
**Variable**	**Serum cadmium (Before the interpolation)**		**Serum cadmium (after the interpolation)**	
	**OR(95% CI)**	***P*** **-value**	**OR(95% CI)**	***P*** **-value**
Model 1	0.990 (0.966–1.016)	0.449	1.007 (0.892–1.136)	0.912
Model 2	1.007 (0.983–1.032)	0.569	0.745 (0.649–0.856)	<0.001
Model 3	1.007 (0.975–1.040)	0.679	0.798 (0.688–0.925)	0.003
Model 4	1.006 (0.970–1.043)	0.743	1.009 (0.982–1.037)	0.511

Model 1: non-adjusted.

Model 2: age, gender, race, education.

Model 3: Model 2+ BMI, WC, smolking, alchol consumption, hypertension.

Model 4: Model 3+ HDL, TG, ALT, GGT, LDH, ALB.

### Curve fitting analysis

**Figure 3:**
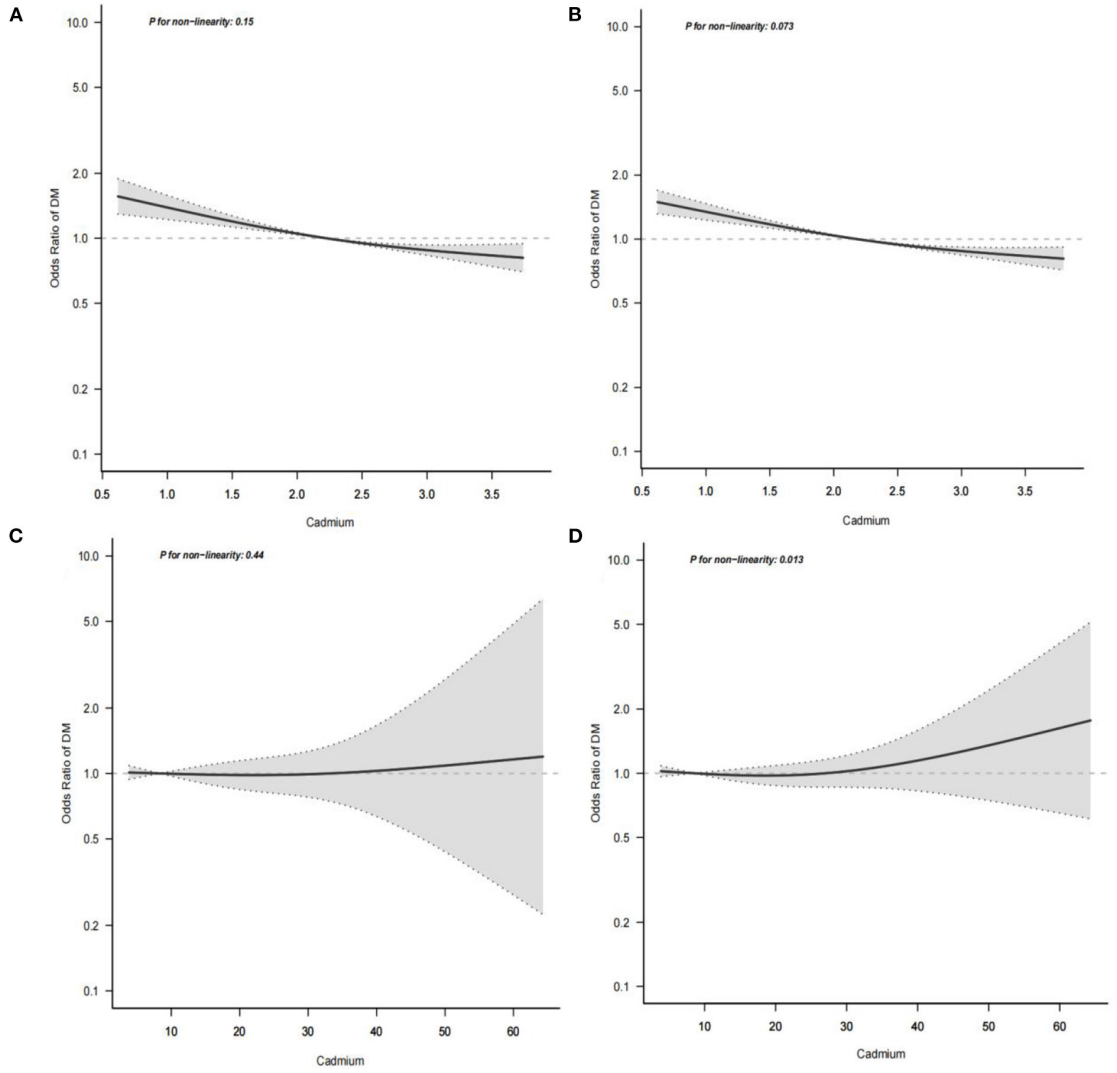
Multivariate logistic regression analysis of the associations of serum cadmium levels with diabetes. The horizontal axis represents the serum cadmium level( mmol/L), and the vertical axis represents the relative probability of developing diabetes. **(A)** Shows the association between serum cadmium level and diabetes in low cadmium exposure group before missing covariates were imputed. **(B)** Shows the association between serum cadmium level and diabetes in low cadmium exposure group after missing covariates were imputed. **(C)** Shows the association between serum cadmium level and diabetes in high cadmium exposure group before missing covariates were imputed. **(D)** Shows the association between serum cadmium level and diabetes in high cadmium exposure group after missing covariates were imputed.

In our study, a smooth curve fitting diagram was drawn to visually describe the relationship between serum cadmium level and diabetes, and the linear relationship was tested. In the low cadmium exposure group, as shown in Figures 3A,B, the association between serum cadmium and diabetes was linear with monotonicity decreasing, and *P* for non-linearity was >0.001, indicating a gradual decrease in the rate of diabetes with increased serum cadmium. In the high cadmium exposure group, the association between serum cadmium level and diabetes was linear with monotonicity and transverse axis parallel, and *P* for non-linearity was >0.001, which indicates that the occurrence of diabetes was not modified by serum cadmium level (Figures 3C,D).

### Subgroup analysis

**Figure 4:**
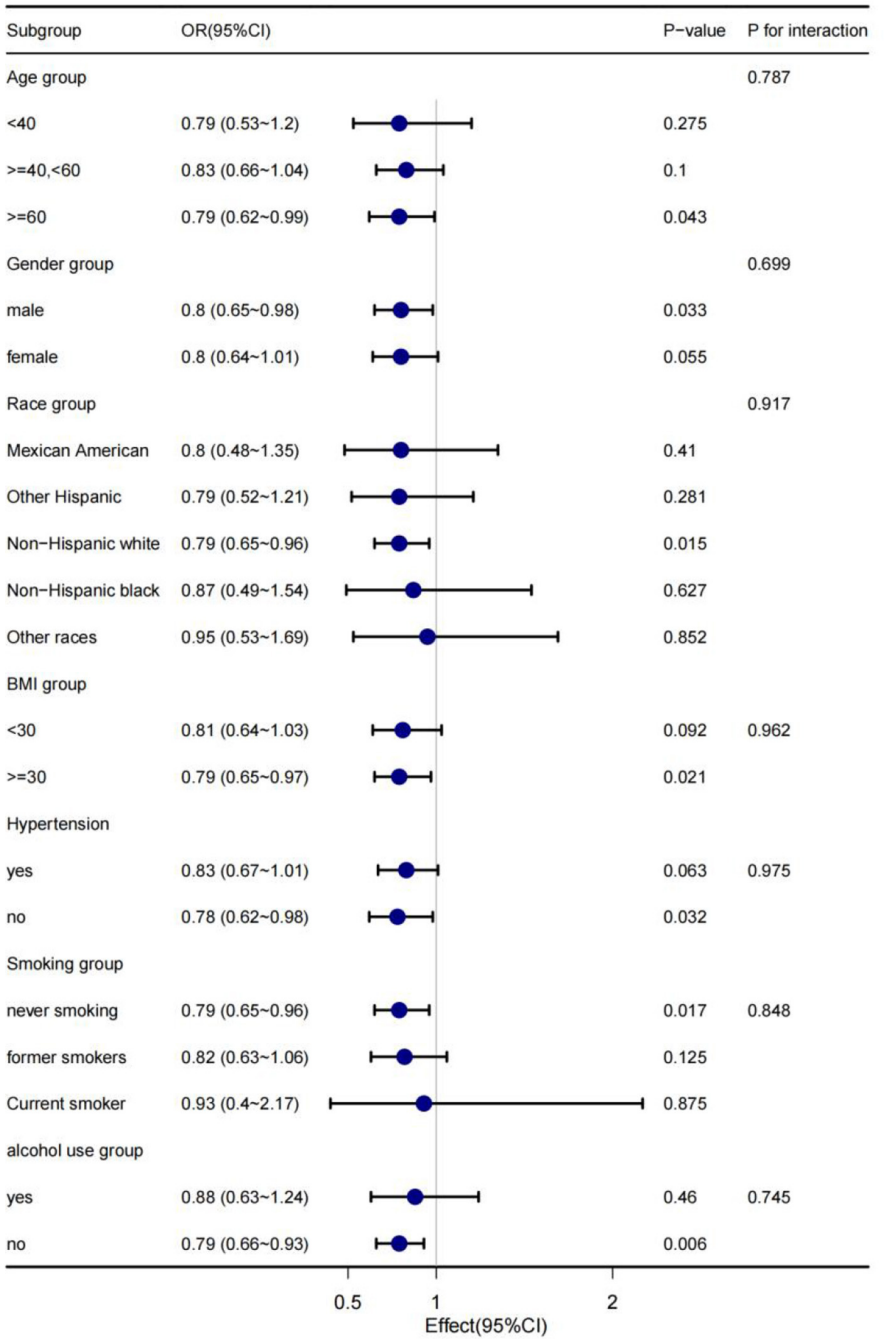
The stratification logistics regression analysis of the associations of serum cadmium levels in low cadmium exposure with diabetes in different subgroup.

**Figure 5:**
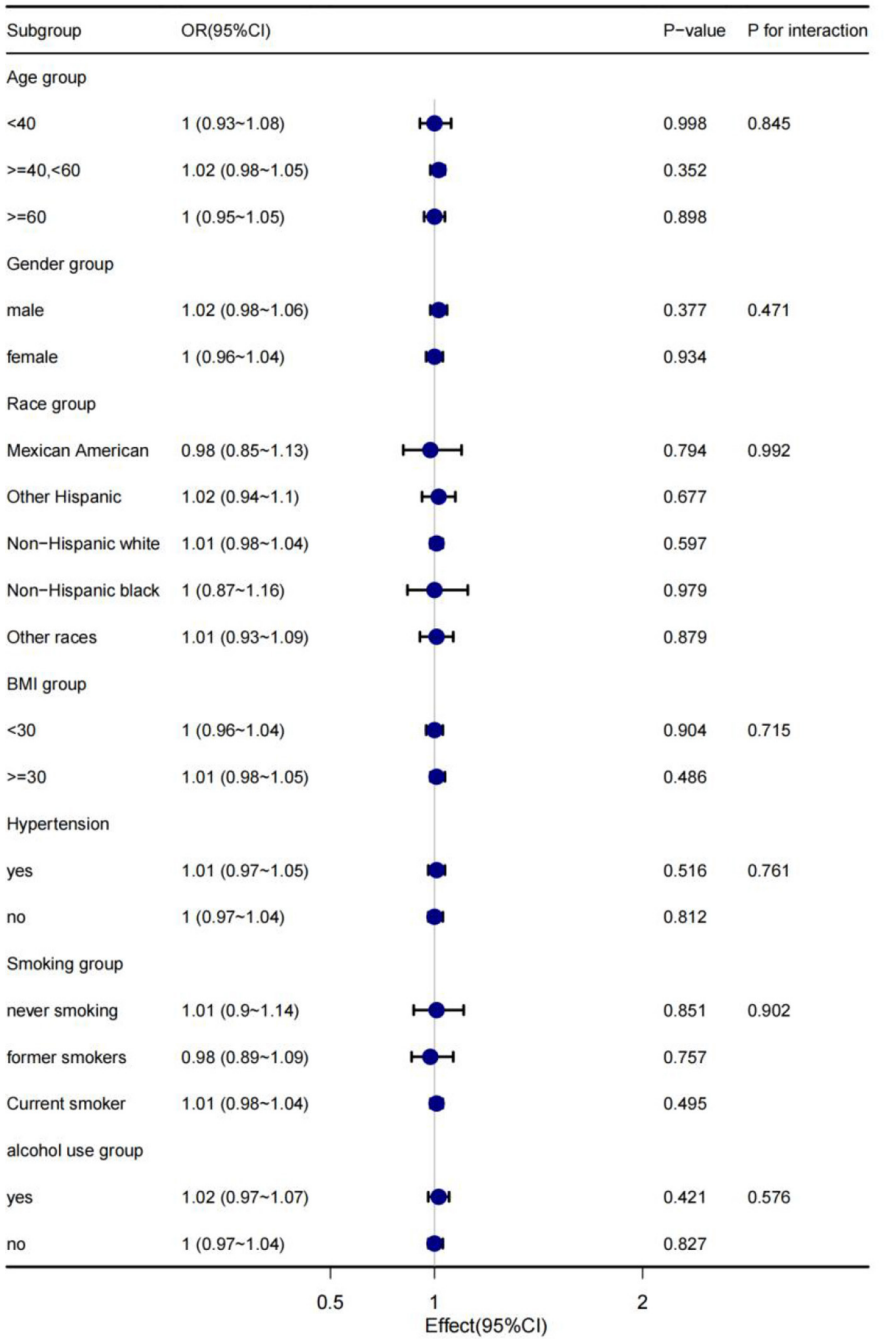
The stratification logistics regression analysis of the associations of serum cadmium levels in high cadmium exposure with diabetes in different subgroups.

In order to explain the results preferably and find out whether there are special groups in the current population, we divided them into groups by age, gender, race, BMI, hypertension, smoking and alcohol consumption, and analyzed them using classified logistics regression. As shown in Figure 4, in the low-cadmium exposure group, the association between serum cadmium levels and diabetes remained stable in all subgroups, including age, sex, race, BMI, hypertension, smoking, and alcohol consumption; the association between serum cadmium levels and diabetes was not present in all subgroups, including age, sex, race, BMI, hypertension, smoking, and alcohol consumption in the high cadmium exposure group (Figure 5).

## Discussion

The present study used data for adults from 11 cycles of NHANES (1999–2020) to explore the association between serum cadmium levels and diabetes. According to the level of serum cadmium of candidates, the study population was divided into low cadmium exposure group and high cadmium exposure group, and it was demonstrated that the association between cadmium and diabetes was not consistent at different exposure levels. After adjusting for potential confounding, there was a negative association between serum cadmium levels and diabetes. The effect value OR and 95% CI were 0.811 (0.698, 0.943), respectively. No association between serum cadmium levels and diabetes was observed in the high cadmium exposure group (*P* > 0.05). The study population was divided into different subgroups according to age, gender, race, BMI, hypertension, smoking and alcohol consumption in subgroup analysis. In these subgroups, the association was stable with no special population being found, meaning that the finding could be generalized to the population. In addition, taking serum cadmium as a categorical variable, we explored the trend of the association between serum cadmium and diabetes, and we got similar conclusions, which further supported our conclusions.

Amounting studies have been performed to explore the association between serum cadmium levels and diabetes, whereas the results are inconsistent and contradictory. Some epidemiological studies suggested that cadmium exposure may be related higher diabetes risk, one cycle of the U.S. population study (NHANES 1988-1994), Schwartz et al. found the prevalence of diabetes in participants positively associated with urinary cadmium levels (29). One recent review and meta-analysis found a positive association between cadmium exposure and risk of diabetes with a dose-response relation and moderate-certainty evidence (28). However, contrary findings were reported, a case-control study (*n* = 876) showed cadmium was significantly negatively associated with diabetes when only adjusted age and sex in participants from the HUNT3 Survey (30). A small part of studies investigating the relation between blood cadmium levels and diabetes showed an irrelevant association. One Chinese study (41) conducted among Chinese adults (*n* = 5,554) observed blood cadmium was positively related to prediabetes, whereas no association was observed between blood cadmium and diabetes. In a Swedish cohort study (*n* = 4,585), Borne et al. Found that elevated blood cadmium levels are not associated with increased incidence of diabetes. A cross-sectional study (32) also showed cadmium exposure was not associated with increased risk of diabetes in older women in Sweden (*n* = 2,595). Nonetheless, taken these studies together, there would be many limitations these studies, such as the sample sizes differ from different studies. There are also differences in the study population. Since the prevalence of diabetes varies among different races, the baseline of each study population and covariates adjusted are different. Moreover, the association is not explored under different exposure levels of serum cadmium. All of limitations above would not be likely to provide sufficient power to demonstrate cadmium exposure effects on diabetes. Since research on the general population about cadmium exposure to diabetes relatively limited, it's of great significance to supplement research in general population in Americ. Based on summarizing the limitations and bias of previous studies, we designed the study and analyzed associations in a high levels of exposure population and low levels exposure population, therefore our study is different from the previous studies.

In the present study, we did not observe significant association between serum cadmium and diabetes in high exposure population, which was consistent with the results from previous epidemiological studies (31, 32). However, it is undeniable that the toxic effect of cadmium on the human body is still apparent when it involves to lung and kidney cancers (42). In addition to cancer, numerous studies suggested that elevated cadmium concentrations have been positively associated with an increased risk of chronic diseases such as cardiovascular diseases and bone disease (43). Therefore, we confirmed that cadmium was a high risk factor for human health at high exposure levels.

Special mention should be made of the result that serum cadmium levels were negatively associated with the occurrence of diabetes in the low serum cadmium exposure group, the incidence of diabetes decreased with the increase of serum cadmium level at values under 3.2 mmol/L. As far as we know, the mechanisms involved are not well defined. We speculated that cadmium might play a physiological role at low levels somewhat, although it has not yet been discovered. Cadmium is chemically similar to zinc since they belong to group 12 of the periodic table and both elements are divalent d-block elements (44). Previous studies have shown that serum zinc levels are negatively correlated with diabetes and have positive glucose control outcomes (45). Zinc is a protective factor for diabetes and supplemental zinc may significantly contribute to the management of diabetic hyperglycemia and related metabolic abnormalities (46). Given this, we boldly speculate that cadmium may play a physiological function similar to zinc at low exposure levels to a certain extent.

It is undeniable that there are several limitations to the present study. Firstly, this study is a cross-sectional study, affected by the inherent defects of the study itself, and causal conclusions are unwarranted. Concerning this, it will be essential to conduct a well-designed cohort study. In addition, in order to explore the association between serum cadmium and diabetes in the general population, this study did not include the special population such as children and pregnant, in the future study, we will try to bring into particular population for analysis. Additionally, due to the limitation of questionnaire information collection and other factors, the study failed to effectively distinguish people with diabetes into type 1 and type 2 diabetes. However, among US adults with diagnosed diabetes, type 1 and type 2 diabetes accounted for 5.6 and 91.2% (47), respectively, considering the high proportion of type 2 diabetes in the United States, it will not have much impact on the conclusion to a certain extent. In addition, there are some limitations for using serum cadmium to assess the population exposure to cadmium compared to other matrices as whole blood and urinary concentrations. In fact, the purpose and content of this cross-sectional study is to observe whether there is a potential association between serum cadmium level with diabetes. The extent to which serum cadmium can reflect cadmium exposure does require further research, more accurate indicator will be used to replace serum cadmium in our future study. The present study also has several strengths. Data used from a large nationally representative sample from NHANES, which used rigorous data collection procedures, and weight analysis was carried out according to the weight method provided by NHANES official website that could increase the statistical power and provide a more reliable and accurate result. The study population was divided into different exposure groups according to the level of serum cadmium,. The exposure of low cadmium was in line with the actual situation of exposure of the normal population, which made our study practical significance. Multiple interpolations were used to interpolate covariates with missing data, which expanded the statistical efficiency.

## Conclusions

Based on the perspective of general population exposure to the environment, our results suggested that under the normal level of serum cadmium exposure, there was a negative association between serum cadmium and diabetes among the representative samples of the whole population in the United States, which layed a solid foundation for environmental scientists and clinical scientists to study the association between cadmium exposure and diabetes in the future. In addition, it is with great potential in practice. However, there was no association between serum cadmium level and the occurrence of diabetes in the high serum cadmium exposure group. Future research using multicenter and prospective cohort study designs would still need to verify these results.

## Data availability statement

Publicly available datasets were analyzed in this study. This data can be found here: All datasets can be downloaded in NHANES website (https://www.cdc.gov/nchs/nhanes/).

## Ethics statement

The authors are accountable for all aspects of the work in ensuring that questions related to the accuracy or integrity of any part of the work are appropriately investigated and resolved. The study was conducted in accordance with the Declaration of Helsinki (as revised in 2013). All information from the NHANES program is available and free for public, so the agreement of the medical ethics committee board was not necessary.

## Author contributions

RG and YW conceived the idea. RG wrote the manuscript. YW and ZC collected, read the literature, and revised the article. ZC collected and collated the data needed for this article. ZC and JD read through and corrected the manuscript. All authors read and approved the final manuscript.
