# Supplementary material for: The association between serum cadmium and diabetes in the general population: A cross-sectional study from NHANES (1999–2020)
Source: Front Nutr. 2022 Dec 7;9:966500. doi: 10.3389/fnut.2022.966500 (PMC9768494; doi:10.3389/fnut.2022.966500)
Supplement: Supplementary file 1 [file Data_Sheet_1.pdf]

Appendix

Supplement Table legends:

Table S1. Serum cadmium levels under high cadmium exposure after multiple interpolation

Table S2. Serum cadmium levels under high cadmium exposure after multiple interpolation

Table S3. Serum cadmium levels as categorical variable under low cadmium exposure after multiple interpolation

Table S4. Serum cadmium levels as categorical variable under high cadmium exposure after multiple interpolation

Table S1. Serum cadmium levels under high cadmium exposure after multiple interpolation

Table S1. Serum cadmium levels under high cadmium exposure after multiple interpolation

Variable	IMP 1		IMP 2		IMP 3		IMP 4		IMP 5	
	OR(95%CI)	*P*-value	OR(95%CI)	*P*-value	OR(95%CI)	*P*-value	OR(95%CI)	*P*-value	OR(95%CI)	*P*-value
Model 1	1.009 (0.894~1.139)	0.881	1.009 (0.894~1.139)	0.883	1.003 (0.889~1.132)	0.955	1.007 (0.892~1.136)	0.912	1.004 (0.889~1.133)	0.954
Model 2	0.742 (0.647~0.852)	<0.001	0.743 (0.647~0.853)	<0.001	0.737 (0.642~0.847)	<0.001	0.745 (0.649~0.856)	<0.001	0.741 (0.645~0.851)	<0.001
Model 3	0.793 (0.684~0.92)	0.002	0.791 (0.682~0.918)	0.002	0.793 (0.683~0.920)	0.002	0.798 (0.688~0.925)	0.003	0.789 (0.680~0.915)	0.002
Model 4	0.806 (0.693~0.936)	0.005	0.805 (0.692~0.936)	0.005	0.806 (0.693~0.937)	0.005	0.811 (0.698~0.943)	0.007	0.803 (0.690~0.934)	0.004

Model 1: non-adjusted

Model 2: age,gender,race,education

Model 3: Model 2+ BMI,waist,smolking,alchol use,hypertension

Model 4: Model 3+ HDL, TG, ALT, GGT, LDH, ALB

Note: IMP represents the interpolated data

Table S2. Serum cadmium levels under high cadmium exposure after multiple interpolation

Table S2. Serum cadmium levels under high cadmium exposure after multiple interpolation

Variable	**IMP** 1		**IMP** 2		**IMP** 3		**IMP** 4		**IMP** 5	
	OR(95%CI)	*P*-value	OR(95%CI)	*P*-value	OR(95%CI)	*P*-value	OR(95%CI)	*P*-value	OR(95%CI)	*P*-value
Model 1	0.99 (0.97~1.02)	0.48	0.99 (0.97~1.02)	0.50	0.99 (0.97~1.01)	0.43	0.99 (0.97~1.02)	0.50	0.99 (0.97~1.02)	0.58
Model 2	1.01 (0.99~1.03)	0.43	1.01 (0.99~1.03)	0.44	1.01 (0.99~1.03)	0.47	1.01 (0.99~1.03)	0.42	1.01 (0.99~1.03)	0.33
Model 3	1.01 (0.98~1.04)	0.49	1.01 (0.98~1.04)	0.48	1.01 (0.98~1.04)	0.47	1.01 (0.98~1.04)	0.46	1.01 (0.99~1.04)	0.37
Model 4	1.01 (0.98~1.04)	0.54	1.01 (0.98~1.04)	0.54	1.01 (0.98~1.04)	0.53	1.01 (0.98~1.04)	0.53	1.01 (0.99~1.04)	0.41

Model 1: non-adjusted

Model 2: age,gender,race,education

Model 3: Model 2+ BMI,waist,smolking,alchol use,hypertension

Model 4: Model 3+ HDL, TG, ALT, GGT, LDH, ALB

Note: IMP represents the interpolated data

Table S3. Serum cadmium levels as categorical variable under low cadmium exposure after multiple interpolation

Table S3. Serum cadmium levels as categorical variable under low cadmium exposure after multiple interpolation

Variable	Model 1		Model 2		Model 3		Model 4	
	OR(95%CI)	*P*-value	OR(95%CI)	*P*-value	OR(95%CI)	*P*-value	OR(95%CI)	*P*-value
Q1	1(Ref)		1(Ref)		1(Ref)		1(Ref)	
Q2	0.89 (0.66~1.19)	0.42	0.74 (0.54~1.01)	0.06	0.82 (0.59~1.15)	0.26	0.85 (0.60~1.19)	0.34
Q3	1 (0.74~1.35)	0.99	0.6 (0.43~0.84)	<0.01	0.69 (0.48~0.98)	0.04	0.72 (0.50~1.03)	0.07
Q4	1.03 (0.76~1.38)	0.86	0.51 (0.37~0.72)	<0.01	0.61 (0.42~0.87)	<0.01	0.64 (0.44~0.92)	0.02
P for trend test	1.02 (0.93~1.12)	0.66	0.80 (0.72~0.89)	<0.01	0.85 (0.75~0.95)	<0.01	0.86 (0.76~0.97)	0.01

Model 1: non-adjusted

Model 2: age,gender,race,education

Model 3: Model 2+ BMI,waist,smolking,alchol use,hypertension

Model 4: Model 3+ HDL, TG, ALT, GGT, LDH, ALB

Table S4. Serum cadmium levels as categorical variable under high cadmium exposure after multiple interpolation

Table S4. Serum cadmium levels as categorical variable under high cadmium exposure after multiple interpolation

Variable	Model 1		Model 2		Model 3		Model 4	
	OR(95%CI)	*P*-value	OR(95%CI)	*P*-value	OR(95%CI)	*P*-value	OR(95%CI)	*P*-value
Q1	1(Ref)		1(Ref)		1(Ref)		1(Ref)	
Q2	0.90 (0.61~1.34)	0.60	0.90 (0.60~1.36)	0.63	0.89 (0.58~1.38)	0.62	0.89 (0.58~1.39)	0.62
Q3	0.86 (0.57~1.28)	0.45	0.91 (0.6~1.4)	0.68	0.90 (0.56~1.45)	0.67	0.88 (0.54~1.42)	0.60
Q4	0.79 (0.54~1.17)	0.24	1.06 (0.7~1.61)	0.78	1.06 (0.63~1.76)	0.83	1.03 (0.61~1.72)	0.91
P for trend test	0.93 (0.82~1.05)	0.24	1.02 (0.89~1.17)	0.78	1.01 (0.86~1.2)	0.87	1 (0.85~1.19)	0.96

Model 1: non-adjusted

Model 2: age,gender,race,education

Model 3: Model 2+ BMI,waist,smolking,alchol use,hypertension

Model 4: Model 3+ HDL, TG, ALT, GGT, LDH, ALB
